# Terahertz Dielectric Characterization and Hybrid Debye–Lorentz Modeling of Silicone Rubber Composites for Composite Insulators

**DOI:** 10.3390/polym18121427

**Published:** 2026-06-08

**Authors:** Tengyi Zhang, Li Cheng, Shuo Zhang, Bo Tao, Qingyue Tan

**Affiliations:** 1 School of Electrical Engineering, Chongqing University, Chongqing 400044, China; 2 State Grid Chongqing Electric Power Company, Chongqing 404100, China

**Keywords:** silicone rubber, dielectric spectroscopy, THz-TDS, Debye–Lorentz model

## Abstract

High-temperature vulcanized (HTV) silicone rubber serves as the core material for composite insulators, and its high-frequency dielectric properties directly dictate its macroscopic insulation performance. However, traditional electrical detection methods encounter a “high-frequency blind zone” above the gigahertz (GHz) range due to limited precision and ambiguous physical mechanisms. In this study, terahertz time-domain spectroscopy (THz-TDS) was employed to characterize the complex permittivity spectra of silicone rubber specimens, incorporated with varying ratios of alumina trihydrate (ATH) and silica (SiO_2_) fillers, across the 0.1–3.0 THz frequency range. Experimental results reveal that the terahertz dielectric characteristics of silicone rubber exhibit a pronounced filler dependency: as the ATH content increases from 95 phr to 185 phr, the real part of the permittivity at 1 THz increases by 32%. Notably, all specimens manifest a sharp dielectric transition near 1.2 THz, characterized by distinct dual absorption peaks in the imaginary permittivity spectra. To characterize this non-linear transition, a hybrid Debye–Lorentz model is innovatively introduced. This approach overcomes the inherent limitations of traditional double Debye models, which are restricted to relaxation processes and fail to account for high-frequency resonance. Fitting results and physical analysis demonstrate that the response at 1.2 THz is primarily attributed to the bending vibrations of Si-O-Si bonds in the polymer backbone, alongside the collective vibration modes of Al-O bonds and the hydrogen-bonded network within the fillers. The hybrid model successfully decouples three distinct polarization mechanisms: conduction loss (<0.5 THz), dipole relaxation (0.5–1.0 THz), and lattice resonance (>1.0 THz). This work provides a robust characterization framework for the quantitative evaluation of the high-frequency dielectric response and microstructural integrity of composite insulators.

## 1. Introduction

High-temperature vulcanized (HTV) silicone rubber serves as a critical external insulation material for composite insulators. Its macroscopic insulation properties, such as dielectric strength and arc resistance, are fundamentally governed by its microscopic dielectric response characteristics [1]. As a fingerprint for characterizing internal charge carrier dynamics and energy dissipation, dielectric spectroscopy provides a direct reflection of the interactions between the polymer matrix and fillers. By investigating the complex permittivity over a broad frequency spectrum, it is possible not only to evaluate the polarization capacity and energy storage density of the material but also to elucidate complex internal relaxation processes through specific loss mechanisms. In the context of ultra-high voltage (UHV) and sophisticated electromagnetic environments, a profound understanding of the dielectric behavior of silicone rubber at extremely high frequencies holds significant academic value for optimizing material formulations and enhancing the stability of charge transport characteristics [2,3].

High-temperature vulcanized (HTV) silicone rubber employs polydimethylsiloxane (PDMS) as its molecular backbone. The flexible Si-O bond structure confers excellent elasticity and insulating characteristics upon the material, while simultaneously enabling a wide array of molecular motions within the system, including segmental motion, side-methyl group rotation, and chain-link twisting or oscillation [4]. These diverse molecular motion modes correspond to differentiated polarization response processes; specifically, the excitation, relaxation, and restoration behaviors of these motional units directly modulate the laws governing charge confinement, migration, and dissipation within the material. Under long-term field service environments characterized by strong electric fields, thermal cycling, moisture, and pollution, composite insulators continuously undergo polymer chain scission, degradation of the cross-linked network, and deterioration of interfacial bonding strength. Such structural damage at the microscopic level progressively alters the intrinsic polarization characteristics and loss levels of the material, ultimately leading to the gradual degradation of overall insulation performance. Meanwhile, the relaxation dynamics of polymers exhibit an exceptionally strong frequency dependence. Slower segmental relaxations and interfacial charge polarization responses are concentrated in the low-frequency region, whereas high-frequency group vibrations and ultrafast molecular relaxations reside in the high-frequency bands [5]. Consequently, relying solely on low-frequency electrical characterization fails to fully capture the full-scale molecular motion mechanisms of the polymer, making it difficult to elucidate the intrinsic correlation between microstructural evolution and the high-frequency dielectric response.

To date, the characterization of dielectric properties in materials has primarily relied on broadband dielectric spectroscopy (BDS) or LCR meters. These conventional techniques typically span a frequency range from microhertz (μHz) to gigahertz (GHz), and are predominantly utilized to investigate dipole orientation polarization, interfacial polarization (the Maxwell–Wagner effect), and low-frequency hopping conduction mechanisms [6]. Thermally stimulated depolarization current (TSDC) spectrometry is a non-destructive characterization technique that analyzes internal trap level distributions, polarization relaxation mechanisms, and charge transport characteristics by measuring the current released during a programmed temperature ramp. However, TSDC inherently reflects only low-frequency and gigahertz (GHz) polarization and charge transport behaviors, failing to cover the high-frequency dielectric regime of 0.1–3 THz [7]. Consequently, TSDC falls short of characterizing microstructural phenomena induced under high-frequency electric fields and impulse conditions, such as lattice vibrations, high-frequency relaxations of molecular chains, and frequency-differentiated loss mechanisms, all of which remain obscured under this thermal-stimulus approach.

However, the ultrafast dynamics of polymer molecular segments—such as picosecond-scale fast relaxation and phonon resonance modes induced by inorganic fillers—possess characteristic frequencies typically situated within the sub-millimeter to micrometer wave regions. Conventional electrical measurement methods are constrained by distributed circuit parameters; as a result, their precision is severely limited upon entering the megahertz (MHz) range and beyond [8]. This creates a high-frequency blind zone in dielectric characterization, precluding the direct detection of microscopic physical processes in the far-infrared band.

Terahertz (THz) technology bridges the gap between electronics and optics, providing a novel dimension for dielectric research. As a non-contact, non-ionizing, and non-destructive characterization modality, THz waves exhibit exceptional sensitivity to polar molecules and lattice vibrations [9]. Compared to Fourier-transform infrared spectroscopy (FTIR), terahertz time-domain spectroscopy (THz-TDS) enables the precise extraction of both the real and imaginary parts of the complex permittivity by directly detecting the amplitude and phase of the electric field signal, thereby bypassing the necessity of the Kramers–Kronig (K-K) transformation [10,11].

Crucially, the THz frequency band encompasses the bending and torsional vibrations of the Si-O-Si backbone in the silicone rubber matrix, as well as the resonance frequencies of filler particles (such as ATH or silica) [12]. Consequently, it serves as a critical spectral window for investigating the transition from relaxation polarization to resonance polarization [13]. Despite the immense potential of THz technology, existing research has primarily focused on static permittivity measurements; there remains a deficiency in in-depth exploration regarding the physical mechanisms of the nonlinear dielectric transition observed in silicone rubber composites above 1 THz [14,15].

To address these gaps, this study employs THz-TDS to acquire the dielectric spectra of HTV within the 0.1–3.0 THz range. Regarding the significant dielectric jump observed near 1.2 THz, this paper innovatively adopts a hybrid Debye–Lorentz model for fitting and analysis. This approach circumvents the inability of traditional dielectric methods to capture high-frequency resonance information and elucidates the influence of filler concentration on the real and imaginary permittivity. Furthermore, by introducing the Debye–Lorentz modification, we overcome the limitations of the single Debye model, which fails within the resonance region. This model physically decouples the contributions of conduction loss, dipole relaxation, and molecular resonance, providing a theoretical and quantitative explanation of the response characteristics and revealing the microscopic polarization essence of silicone rubber composites under high-frequency electromagnetic fields.

## 2. Materials and Methods

### 2.1. Materials and Processing

The silicone rubber (SIR) composites investigated in this study are specifically designed for the housing and sheds of composite insulators. The polymer matrix is based on polydimethylsiloxane (PDMS). To achieve the required mechanical and electrical performance, aluminum trihydrate (ATH) and fumed silica (SiO_2_) were employed as the primary functional and reinforcing fillers, respectively as shown in Table 1. Furthermore, various auxiliary additives were incorporated into the formulation, including hydroxy-terminated silicone oil (as a structure control agent), silane coupling agents (to improve filler–matrix interfacial adhesion), and organic colorants.

The preparation of the SIR samples followed a rigorous multi-stage mechanical compounding and thermal vulcanization protocol to ensure optimal filler dispersion and cross-linking density [16,17]:

1. Mechanical Compounding: The raw PDMS gum was first plasticized in a high-shear internal mixer. ATH and SiO_2_ fillers were incrementally added to the matrix under controlled shear conditions to prevent excessive heat buildup while ensuring the breakdown of filler agglomerates.
2. Milling and Homogenization: The compound was then transferred to a two-roll mill. Through repeated shearing and folding, the additives—including hydroxy silicone oil and coupling agents—were uniformly distributed.
3. Heat Treatment: To eliminate volatile low-molecular-weight components and stabilize the filler–matrix interface, the mixture underwent a vacuum heat treatment process at approximately 150–170 °C.
4. Vulcanization: Finally, after the addition of a peroxide curing agent, the compound was molded into sheets using a high-pressure flat vulcanizing press. The curing was performed at 170 °C and 10 MPa for a duration sufficient to ensure complete cross-linking.

The composition and content of silicone rubber sample filler are shown in Table 2.

The resulting specimens were fabricated into square plates with dimensions of 100 mm × 100 mm and a uniform thickness of 2 ± 0.1 mm. Owing to the transmission configuration employed in this study, the scattering effects induced by surface roughness are minimal and exert no distinct influence on the detected signals. To systematically investigate the influence of filler loading on the dielectric and material properties, a series of SIR samples with varying filler weight ratios were customized in collaboration with a professional composite insulator manufacturer.

### 2.2. THz-TDS Experimental Platform

**Figure 1:**
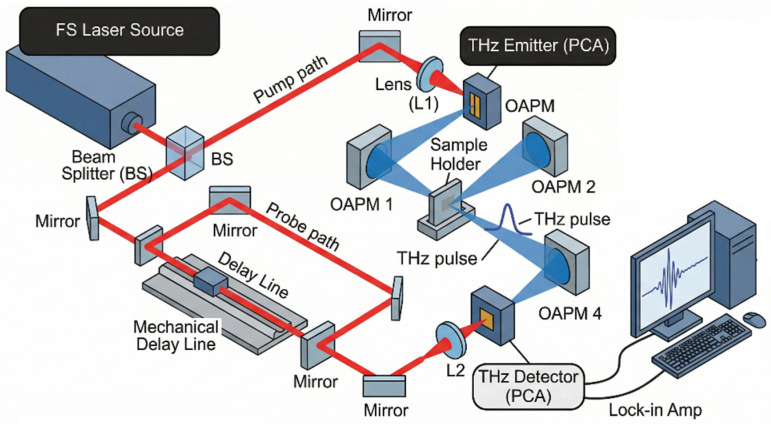
Schematic of the THz-TDS system.

In this study, a typical transmission-mode terahertz time-domain spectroscopy (THz-TDS) system was employed, with its optical layout illustrated in Figure 1. The system primarily consists of a femtosecond laser source, an optical delay line, a THz emitter, a THz detector, and a lock-in amplifier. A fiber-coupled femtosecond laser was utilized as the excitation source, featuring a central wavelength of 1500 nm, a repetition rate of 80 MHz, and a pulse width of less than 100 fs.

The laser output is split into a pump beam and a probe beam by a beam splitter (BS). The pump beam is employed to excite the THz emitter, while the probe beam is used to sample the THz electric field at the detector end. Photoconductive antennas (PCAs) serve as both the THz source and the detector. Specifically, the pump beam irradiates the biased PCA to generate a transient current, thereby radiating THz pulses. Simultaneously, the probe beam modulates the carrier concentration within the detection antenna, allowing for the reconstruction of the THz electric field intensity by measuring the magnitude of the induced photocurrent. The optical collimation system comprises four off-axis parabolic mirrors (OAPMs) configured in a 4f geometry. The first pair of mirrors is responsible for collimating and focusing the THz beam onto the sample center, while the subsequent pair re-collects the transmitted THz radiation and focuses it onto the detection antenna.

The terahertz measurements were performed using a THz-TDS spectrometer (XS-FL1560-30/30-2, Xingsheng Optoelectronics Co., Ltd., Shanghai, China), which integrated commercial photoconductive antennas (TERA15-TX-FC, Menlo Systems, Boulder, CO, USA).

Prior to characterization, the samples were dried in an oven at 65 °C for 30 min. The measurements were conducted within an acrylic enclosure purged with nitrogen gas N_2_, where each recovered signal was averaged over 128 scans to ensure a high signal-to-noise ratio. To mitigate experimental errors, five distinct points were tested on each sample. In this study, transmission-mode terahertz time-domain spectroscopy (THz-TDS) was employed to evaluate the high-temperature vulcanized (HTV) silicone rubber specimens with a thickness of 2 mm, spanning a frequency range of 0.1 to 3.0 THz. Because the specimen thickness is well-optimized, the Fabry–Pérot interference generated at the upper and lower interfaces, alongside the multiple reflections propagating back and forth within the material, exhibited extremely low amplitudes. These optical artifacts caused no discernible distortion in either the time-domain waveforms or the frequency-domain dielectric spectra. Consequently, the interference from these two optical phenomena can be safely neglected during data processing and parameter extraction, ensuring that the experimental results faithfully reflect the intrinsic high-frequency dielectric response characteristics of the material.

### 2.3. THz Data Processing Methods

In this study, terahertz time-domain spectroscopy (THz-TDS) was utilized to characterize the dielectric properties of the composites. The data processing procedure follows a systematic transformation from the time domain to the frequency domain, facilitating the subsequent extraction of the complex refractive index and complex permittivity. Initially, a Fast Fourier Transform (FFT) is applied to convert the experimentally measured reference time-domain electric field, $E_{\mathrm{ref}}(t)$, and sample time-domain electric field, $E_{\mathrm{sam}}(t)$, into the frequency domain. This transformation yields the corresponding complex electric field spectra which can be expressed as:

(1) $$\tilde{E}\left(\omega \right)=\int E\left(t\right)e^{-i\omega t}dt=A\left(\omega \right)e^{i\phi \left(\omega \right)}$$

where ω denotes the angular frequency, A(ω) represents the amplitude spectrum, and ϕ(ω) is the phase spectrum. Consequently, the complex transmission function of the specimen, $\tilde{T}(\omega )$, can be derived as follows:

(2) $$\tilde{T}\left(\omega \right)=\frac{{\tilde{E}}_{\mathrm{sam}\;}\left(\omega \right)}{{\tilde{E}}_{\mathrm{ref}\;}\left(\omega \right)}=\rho \left(\omega \right)e^{i\Delta \phi \left(\omega \right)}$$

where $\rho (\omega )=A_{\mathrm{sam}}/A_{\mathrm{ref}}$ is the amplitude attenuation ratio and $\Delta \phi (\omega )=\phi_{\mathrm{sam}}-\phi_{\mathrm{ref}}$ is the phase difference. For a homogeneous plane-parallel specimen with a thickness of d, the relationship between the complex transmission function and the complex refractive index can be simplified by neglecting multiple reflection effects as follows:

(3) $$\tilde{T}\left(\omega \right)=\frac{4\tilde{n}}{\left(\tilde{n}+1{)}^{2}\right.}\exp\left[i\left(\tilde{n}-1\right)\frac{\omega d}{c}\right]$$

By equating the real and imaginary parts of the aforementioned equations, the refractive index n(ω) and the extinction coefficient κ(ω) of the specimen can be derived as follows:

(4) $$n\left(\omega \right)=1+\frac{c}{\omega d}\Delta \phi \left(\omega \right)$$

(5) $$\kappa \left(\omega \right)=\frac{c}{\omega d}ln\left[\frac{4n\left(\omega \right)}{\rho (\omega )(n(\omega )+1{)}^{2}}\right]$$

According to the propagation characteristics of electromagnetic waves in media as derived from Maxwell’s equations, the complex permittivity $\tilde{\varepsilon }(\omega )$ and the complex refractive index $\tilde{n}(\omega )$ satisfy the following fundamental relationship:

(6) $$\varepsilon^{\prime }(\omega )-i\varepsilon^{\prime \prime }(\omega )=(n-i\kappa {)}^{2}=\left(n^{2}-\kappa^{2}\right)-i\left(2n\kappa \right)$$

Consequently, the expressions for calculating the real and imaginary parts of the permittivity are derived as follows:

(7) $$\varepsilon^{\prime }\left(\omega \right)=n^{2}\left(\omega \right)-\kappa^{2}\left(\omega \right)$$

(8) $$\varepsilon^{\prime \prime }\left(\omega \right)=2n\left(\omega \right)\kappa \left(\omega \right)$$

## 3. Results and Discussion

### 3.1. Evolution of Dielectric Spectra with Filler Content

As a highly cross-linked polymer composite, the dielectric response of silicone rubber in the terahertz (THz) frequency range is collectively driven by multiple microscopic mechanisms. The polymer backbone consists of Si-O-Si bonds, while the side chains typically comprise functional groups such as methyl groups. Under the influence of a THz electric field, although the long-range micro-Brownian motion of the polymer chains is frozen due to the high-frequency nature of the field and the cross-linked network, the localized motion of chain segments and the orientation polarization of side-chain dipoles remain capable of synchronizing with the high-frequency field oscillations. Furthermore, due to the incorporation of reinforcing fillers such as fumed silica (white carbon black), complex interfacial structures are formed between the filler particles and the polymer matrix.

**Figure 2:**
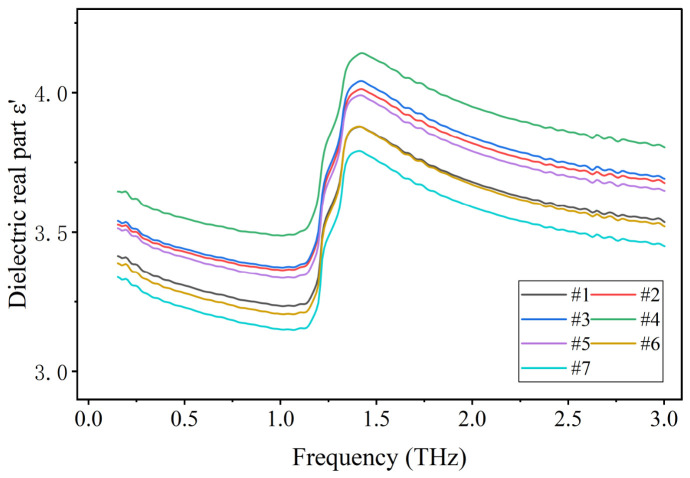
Real part of the permittivity for silicone rubber specimens.

Figure 2 illustrates the frequency-dependent complex permittivity curves for the silicone rubber specimens within the 0.1–3.0 THz range. As shown in Figure 2, the real part of the permittivity $\varepsilon^{\prime }$ for all specimens exhibits a pronounced frequency dispersion effect across the measured band. With the frequency increasing from 0.1 THz to 3.0 THz, an overall upward trend is observed, with values gradually ascending from approximately 2.7 to over 3.5. Notably, a sharp dielectric jump occurs near 1.2 THz, reaching a peak at about 1.4 THz, followed by a gradual decline. This phenomenon of increasing permittivity with frequency typically indicates the presence of a strong resonant absorption peak within this frequency region.

**Figure 3:**
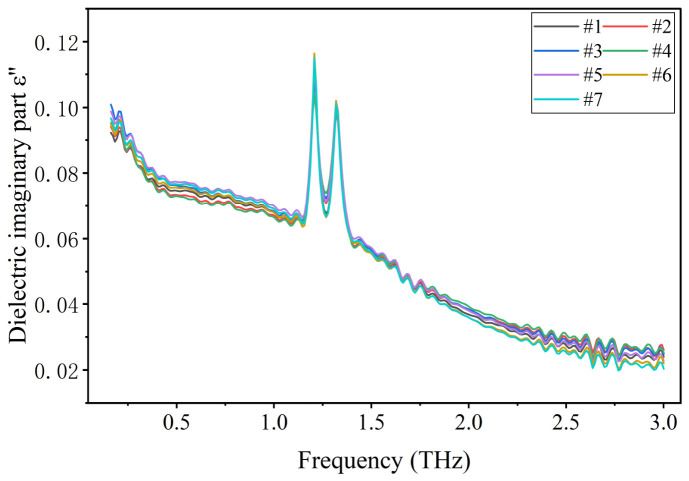
Imaginary part of the permittivity for silicone rubber specimens.

Figure 3 illustrates the dielectric loss characteristics of the specimens. The experimental results indicate that at the low-frequency end (<0.5 THz), the imaginary permittivity $\varepsilon^{\prime \prime }$ remains at a relatively high level, ranging from approximately 0.08 to 0.10. Subsequently, $\varepsilon^{\prime \prime }$ exhibits a gradual decline and tends to stabilize as the frequency increases, before manifesting two distinct dual characteristic absorption peaks in the vicinity of 1.21 THz and 1.32 THz. Such intense fluctuations within a narrow frequency band deviate from the smooth relaxation processes typically observed in lower frequency ranges, representing a signature of resonant characteristics. By 2.8 THz, the value recedes to approximately 0.02. This evolution trajectory of the imaginary part suggests significant energy dissipation processes within the material across the 0–3 THz band.

Under high-frequency terahertz electric fields, while Maxwell–Wagner polarization at the interfaces attenuates, the contributions from dipole responses within the interfacial layers and atomic polarization inside the filler particles remain significant. The upward trend of the real permittivity $\varepsilon^{\prime }$ is primarily attributed to the spectral approximation toward high-frequency normal vibration modes. Meanwhile, the evolution of $\varepsilon^{\prime \prime }$ reflects the relaxation loss incurred by dipoles as they overcome viscous frictional resistance during reorientation [18].

In the vicinity of the resonance frequency, the polarization response cannot instantaneously synchronize with the oscillations of the high-frequency electric field. Consequently, the real part $\varepsilon^{\prime }$ follows an anomalous dispersion trajectory—peaking and subsequently receding—which elucidates the dielectric signal jumps observed in the 1.2–1.5 THz region. Within the 1.2–1.4 THz range, the bending vibrations of the Si-O-Si backbone and the torsional vibrations of long chain segments undergo resonance, leading to intense energy absorption at specific frequencies and the formation of absorption peaks. Furthermore, the fumed silica nanoparticles incorporated into the silicone rubber possess specific phonon vibrational modes in the THz band. The jump near 1.2 THz likely encompasses contributions from collective vibrational modes at the filler–matrix interfaces.

Although the region above 1.2 THz exhibits strong resonant behavior, the dielectric response in the lower terahertz range (0.1–1.0 THz) remains dominated by relaxation processes. To facilitate an in-depth quantitative analysis of the internal charge dynamics, qualitative descriptions alone are insufficient to distinguish between polarization mechanisms at different scales. A single Debye model is inadequate for accommodating these two types of responses with vastly disparate time constants. Moreover, the transition at 1.2 THz serves as the demarcation point between the relaxation and resonance regions. By employing a double Debye model (or a hybrid model), the background relaxation loss can be effectively decoupled from the structural vibrational loss, allowing for the extraction of the static permittivity and relaxation time that reflect the microstructural integrity of the material.

### 3.2. Double Debye Model Fitting and Analysis

Given the coexistence of multiple polarization mechanisms within the material—such as polymer segmental motion and filler/matrix interfacial polarization—the Double Debye model is employed to characterize the complex internal dielectric relaxation processes, as expressed in Equation (9):

(9) $$\varepsilon^{*}\left(\omega \right)=\varepsilon_{\infty }+\frac{\Delta \varepsilon_{1}}{1+i\omega \tau_{1}}+\frac{\Delta \varepsilon_{2}}{1+i\omega \tau_{2}}$$

where $\varepsilon_{\infty }$ is the high-frequency limit permittivity; $\Delta \varepsilon_{1}$ and $\Delta \varepsilon_{2}$ represent the dielectric increments of the respective polarization processes; and $\tau_{1}$ and $\tau_{2}$ denote the polarization relaxation times. Nevertheless, when characterizing silicone rubber composites, the contribution of conduction loss at low frequencies must be explicitly incorporated. The modified expression is given by:

(10) $$\varepsilon^{*}\left(\omega \right)=\varepsilon_{\infty }+\sum_{j=1}^{2}\;\frac{\Delta \varepsilon_{j}}{1+i\omega \tau_{j}}-i\frac{\sigma_{dc}}{\varepsilon_{0}\omega }$$

where $\sigma_{dc}$ denotes the DC conductivity. To extract the dielectric parameters, a global non-linear least-squares fitting was performed based on the double Debye model incorporating the DC conduction term, as expressed in Equations (11) and (12):

(11) $$\varepsilon^{\prime }\left(\omega \right)=\varepsilon_{\infty }+\sum_{j=1}^{2}\;\frac{\Delta \varepsilon_{j}}{1+i\omega \tau_{j}}-i\frac{\sigma_{dc}}{\varepsilon_{0}\omega }$$

(12) $$\varepsilon^{\prime \prime }=\sum_{j=1}^{2}\;\frac{\Delta \varepsilon_{j}\omega \tau_{j}}{1+{\left(\omega \tau_{j}\right)}^{2}}+\frac{\sigma_{dc}}{\omega \varepsilon_{0}}$$

**Figure 4:**
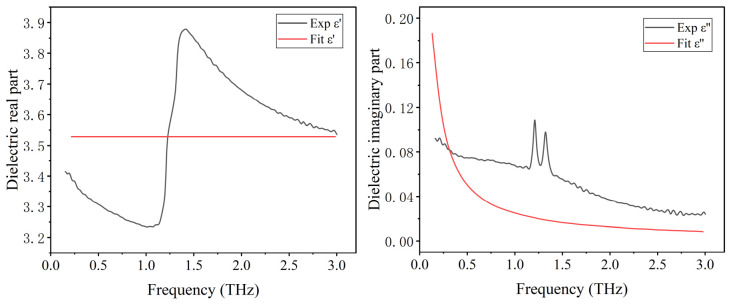
Fitting results of the Double Debye model for the silicone rubber specimens.

The fitting results are presented in Figure 4 and summarized in Table 3.

The extracted high-frequency limit permittivity $\varepsilon_{\infty }$ is approximately 3.5, which is significantly higher than that of the pure silicone rubber matrix. This elevation indicates that the incorporation of inorganic fillers (ATH and SiO_2_) substantially enhances the instantaneous polarization capacity of the composite. Specifically, the ionic displacement polarization of polar bonds within the inorganic particles remains the dominant mechanism in the THz frequency range. The fitting results reveal that the characteristic parameters (corresponding to the resonance frequency) are situated within the 60–80 fs, aligning with the observed resonance near 1.24 THz. The experimental imaginary permittivity $\varepsilon^{\prime \prime }$ exhibits an increase-then-decrease trajectory in the 1.0–3.0 THz band, which is consistent with the predicted position of the absorption peak. This process is primarily attributed to the hydrogen-bonded vibrations of the hydroxyl (-OH) groups in ATH and the interfacial resonance between the SiO_2_ particles and the polymer matrix. The combined dielectric increment $\Delta \varepsilon_{1}$ + $\Delta \varepsilon_{2}$ reaches approximately 4, suggesting that dipole orientation and interfacial relaxation intensities are exceptionally high in this band, serving as the primary sources of energy dissipation.

At frequencies below 0.8 THz, $\varepsilon^{\prime \prime }$ manifests a pronounced declining trend, which is in good agreement with the fitted DC conductivity $\sigma_{dc}$ of approximately 0.8. This suggests that in the low-frequency region, the conduction loss—originating from free carrier motion and interfacial charge accumulation—dominates the dielectric response. As the frequency increases, the charge displacement can no longer synchronize with the rapidly oscillating electric field, leading to a swift attenuation of this loss component.

Furthermore, the inherent limitations of the Debye model must be addressed. By definition, the Debye model describes an overdamped process in dielectrics, where the real part of the permittivity $\varepsilon^{\prime }$ can only decrease monotonically with increasing frequency. Consequently, the Double Debye model is physically incapable of reconstructing the dielectric jump observed at 1.2 THz. By introducing the Lorentz resonance term, the fitting framework transitions from a purely relaxation-based model to an oscillator-type response model. This modification provides a robust physical basis for characterizing the rapid dielectric transitions and anomalous dispersion in the vicinity of 1.2 THz.

### 3.3. Debye–Lorentz Model Fitting and Analysis

**Figure 5:**
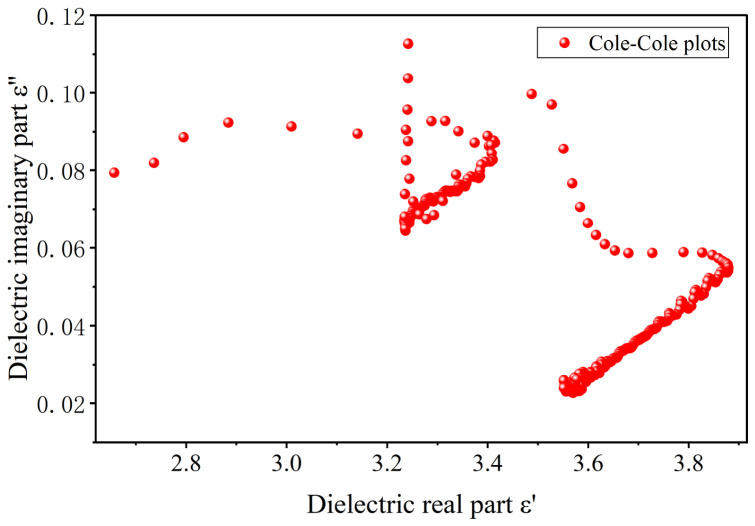
Cole–Cole plots of the silicone rubber specimens.

Within the THz frequency range, it is often challenging to discern the fundamental nature of polarization mechanisms by relying solely on frequency response curves. Instead, the polarization types can be effectively distinguished by analyzing the Cole–Cole plots of the silicone rubber specimens, as illustrated in Figure 5.

In the complex plane plot (Cole–Cole plot), the curve manifests a semicircular arc on the right side (lower-frequency region), which corresponds to the dielectric relaxation of the material. In contrast, an inwardly looping feature appears in the high-frequency region on the left, signifying the presence of resonant modes in the silicone rubber within the 0–3 THz band.

During the relaxation process, the dipoles lag behind the oscillating electric field, and this phase lag gradually accumulates. However, in the resonance region near 1.2 THz, the oscillator amplitude surges dramatically, accompanied by an abrupt 180° phase inversion. This violent shift in polarization intensity causes the real permittivity $\varepsilon^{\prime }$ to first increase and then rapidly diminish—a phenomenon known as anomalous dispersion. In the complex plane, this physical transition is represented by an inward-sweeping trajectory that departs from the standard Debye relaxation path.

The response of silicone rubber near 1.2 THz is typically associated with the torsional vibrations of the polymer backbone, the bending vibrations of the silicon–oxygen bonds Si-O-Si, and the synergistic effects of the ATH fillers [19]. To physically account for these features, it is imperative to incorporate a Lorentz resonance term into the fitting framework. The hybrid Debye–Lorentz model is expressed as follows:

(13) $$\varepsilon^{*}(\omega )=\varepsilon_{\infty }+\sum_{j=1}^{2}\;\frac{\Delta \varepsilon_{j}}{1+i\omega \tau_{j}}+\frac{\Delta \varepsilon_{lor}\omega_{0}^{2}}{\omega_{0}^{2}-\omega^{2}-i\omega \gamma }$$

The newly introduced parameters in the model include the resonant angular frequency $\omega_{0}$, which corresponds to the characteristic vibrational mode observed at 1.2 THz; the damping factor γ, which reflects the linewidth of the resonance peak; and the oscillator strength $\Delta \varepsilon_{lor}$, representing the magnitude of the resonant polarization.

Regarding the fitting results, Real Part: Coefficient of determination R^2^ = −0.66; Pearson correlation coefficient r = −0.93 (with r^2^ = 0.871). Imaginary Part: Coefficient of determination R^2^ = −0.846; Pearson correlation coefficient r = −0.872 (with r^2^ = 0.612). The global R^2^ values are negative, indicating an inadequate absolute quantitative match. In mathematical statistics, a negative R^2^ occurs when the sum of squared residuals of the model exceeds that of a simple horizontal line representing the data mean. In our case, this is primarily because the real-world high-temperature vulcanized (HTV) silicone rubber composite possesses an extremely complex dielectric background. The macroscopic background baseline shifts due to the overlapping tails of low-frequency interfacial polarization and higher-frequency (>3 THz) lattice vibrations. Because our single-resonance Debye–Lorentz model is highly simplified and does not fully deconvolve these extensive off-resonance backgrounds, the optimization algorithm sacrificed global baseline alignment to capture the sharp feature at 1.2 THz, resulting in a large residual sum of squares and negative R^2^ values.

Crucially, while R^2^ reflects the absolute numerical proximity, the Pearson correlation coefficient r reflects the linear alignment of spectral trends and shapes. The squares of the correlation coefficients are remarkably high (r^2^ = 0.871 for the real part and 0.612 for the imaginary part). This statistically demonstrates that the hybrid Debye–Lorentz model is highly sensitive and successful in reproducing the geometric topology of the spectral anomaly, the abrupt jump and peak structures at 1.2 THz. The negative sign of r indicates a phase/direction inversion during global constraint, but the high r^2^ validates that the mathematical expression of a Lorentz oscillator is inherently the correct physical framework to describe this localized phenomenon.

**Figure 6:**
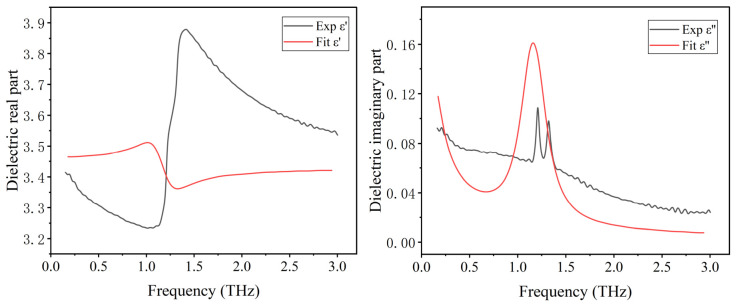
Fitting results of the hybrid Debye–Lorentz model for the silicone rubber composites.

While the traditional Double Debye model is restricted to fitting a smooth, monotonically declining curve near 1.2 THz, the hybrid Debye–Lorentz model successfully reconstructs the discrete “jump” structure observed in the dielectric spectra. This achievement validates the physical fidelity of the hybrid model in characterizing the underlying polarization mechanisms. As illustrated in Figure 6, the real part of the permittivity $\varepsilon^{\prime }$ fitted by the Debye–Lorentz model manifests a distinct transition at 1.2 THz, accompanied by a sharp resonant peak in the imaginary part $\varepsilon^{\prime \prime }$.

In the terahertz frequency regime, the energy of THz radiation primarily corresponds to intermolecular forces and the collective vibrations of polymer chains. Given that silicone rubber consists of siloxane polymers and inorganic fillers, the resonance at 1.2 THz is predominantly attributed to the vibrational modes of the Si-O-Si backbone and the interfacial resonance within the filler–matrix regions.

The Debye model is fundamentally designed to describe overdamped processes in dielectrics. In contrast, the incorporation of the Lorentz term enables the model to capture resonant processes. Near 1.2 THz, the characteristic fluctuation of $\varepsilon^{\prime }$—rising and subsequently falling—represents the anomalous dispersion of the medium. When the frequency of the external electromagnetic wave approaches the intrinsic vibrational frequency of the molecules, the phase difference of the molecular oscillations undergoes a drastic shift. The absorption peak in $\varepsilon^{\prime \prime }$ at 1.2 THz signifies resonant absorption: at this specific frequency, the THz energy is efficiently converted into the kinetic energy of molecular vibrations, which is eventually dissipated as thermal energy through intermolecular collisions. In the mathematical model, this energy dissipation is represented by the imaginary component.

The fitting results, as summarized in Table 4, reveal a prominent Lorentz resonance peak at 1.2 THz, which demonstrates a high degree of consistency with the experimental trend of the imaginary permittivity $\varepsilon^{\prime \prime }$. This resonance frequency is primarily attributed to the bending vibrations of Al-O bonds within the ATH crystal structure and the collective vibration modes of the hydrogen-bonded network formed by hydroxyl (-OH) groups. The incorporation of SiO_2_ likely further broadens the absorption spectrum in this frequency band through interfacial coupling effects.

The extracted damping factor γ of approximately 0.27 reflects the energy dissipation rate of the forced oscillators within the silicone rubber matrix. Due to the robust interaction between the inorganic filler particles ATH and SiO_2_ and the polymer segments, the interface serves as a scattering site for phonon propagation. This interaction results in frequency-selective dissipation rather than ideal simple harmonic oscillation.

Furthermore, the fitted DC conductivity value of approximately 1 indicates that at the onset of the THz spectrum, the dielectric response remains influenced by microscopic conductivity. As frequency increases, the sharp decline in the imaginary part provides evidence that classical conduction loss gradually recedes due to restricted carrier mobility, subsequently being replaced by the high-frequency resonance loss described by the Lorentz term.

The introduction of the Lorentz term also effectively elucidates the subtle variations observed in the real permittivity $\varepsilon^{\prime }$. Prior to reaching the resonance frequency of 1.2 THz, the phase of the Lorentz oscillator lags behind the external electric field, thereby contributing additional polarization capacity. As the filler loading increases, this resonance-induced polarization enhancement superimposes with the intrinsic high-dielectric characteristics of the fillers. Together, these factors lead to the steady decline of the real part across the 0–3 THz range.

## 4. Conclusions

In this study, the terahertz dielectric properties of composite insulator silicone rubber were investigated using THz-TDS within the 0.1–3.0 THz range. By employing a hybrid Debye–Lorentz model, the complex polarization mechanisms and the unique dielectric jump at 1.2 THz were quantitatively analyzed. The main conclusions are as follows:

1. With aluminum trihydroxide (ATH) filler loading rising from 95 phr to 185 phr, the dielectric constant of samples increases monotonically by 32% at 1 THz, which is governed by two microscale mechanisms. First, ATH possesses a far higher intrinsic dielectric constant than silicone rubber matrix; increasing the fraction of this high-dielectric filler raises the overall equivalent dielectric performance of composites. Second, more filler particles generate abundant heterogeneous interfaces between filler and matrix. Under terahertz (THz) electric excitation, charges accumulate and align at these interfaces, enhancing interfacial polarization and dielectric constant. Meanwhile, uniform filler dispersion alters the conformation and mobility of siloxane molecular chains, creating more active charge sites for polarization. These factors jointly improve dielectric properties as filler content grows. This variation provides a reliable basis for regulating the high-frequency dielectric performance of composite insulators via filler modification.
2. All ATH-filled silicone rubber composites exhibit a stable dielectric abrupt transition near 1.2 THz. This is an intrinsic high-frequency electrical property of the material system, independent of filler proportion. From the perspective of molecular and crystalline vibration, the transition originates from collective resonance of internal chemical bonds. The polydimethylsiloxane (PDMS) matrix is rich in Si-O-Si bonds whose vibration frequencies fall around 1.2 THz, while ATH crystals contain numerous Al-O bonds resonating within the same band. The coupled vibrations of the two bond types switch the material’s polarization mechanism from low-frequency dipole relaxation to lattice resonance, presenting as a sharp change in macroscopic dielectric parameters. It proves that matrix molecular structure and filler crystal characteristics jointly determine the material’s high-frequency dielectric rules.

The proposed hybrid Debye–Lorentz model distinguishes three polarization behaviors and band-dependent dominant loss mechanisms: conduction loss prevails below 0.5 THz, dipole relaxation dominates in 0.5–1.0 THz, and lattice resonance becomes prominent above 1.0 THz. The model supports quantitative research on high-frequency dielectric responses. As a promising non-destructive testing technique, terahertz time-domain spectroscopy (THz-TDS) can be further applied to detect microstructural defects and filler dispersion in composite insulators in follow-up studies.

## Figures and Tables

**Table 1:** Information on related materials.

Name	CAS Numbers	Supplier	Specifications	Purity
184 Silicone	/	Dow Corning Co., Ltd. (Midland, MI, USA)	Weight-average (M_v_) 25,000	/
ATH	21645−51−2	Shanghai Yien Chemistry Technology Co., Ltd. (Shanghai, China)	5000 mesh	AR
SiO_2_	14464−46−1	Shanghai Yien Chemistry Technology Co., Ltd. (Shanghai, China)	12,500 mesh	AR

**Table 2:** Formulations of the silicone rubber specimens.

Sample ID	PDMS (phr)	ATH Filler (phr)	SiO_2_ (phr)	Additives (phr)
1	100	95	35	10
2	100	125	35	10
3	100	155	35	10
4	100	185	35	10
5	100	155	15	10
6	100	155	25	10
7	100	155	45	10

**Table 3:** Dielectric parameters extracted from the Double Debye model.

Sample	$\varepsilon_{\infty }$	$\Delta \varepsilon_{1}$	$\tau_{1}$	$\Delta \varepsilon_{2}$	$\tau_{2}$	$\sigma_{dc}$
1	3.53	2.09	62.71	2.03	62.71	0.80
2	3.66	2.18	68.89	2.12	68.89	0.82
3	3.68	2.55	67.04	2.49	67.04	0.88
4	3.79	2.39	82.16	2.34	82.16	0.85
5	3.63	2.59	75.74	2.54	75.74	0.94
6	3.51	2.07	59.73	2.01	59.73	0.79
7	3.44	2.16	53.58	2.10	53.58	0.76

**Table 4:** Dielectric parameters extracted from the hybrid Debye–Lorentz model fitting.

Sample	$\epsilon_{\infty }$	$\Delta \varepsilon_{1}$	$\tau_{1}$	$\Delta \varepsilon_{2}$	$\tau_{2}$	$\omega_{0}$	γ	$\sigma_{dc}$
1	3.43	5.00	626.14	5.00	626.14	1.18	0.27	1.00
2	3.54	5.00	622.77	5.00	622.77	1.18	0.27	1.00
3	3.70	5.00	620.22	5.00	620.21	1.16	0.33	1.00
4	3.81	5.00	662.16	5.00	662.16	1.16	0.34	1.00
5	3.58	5.00	622.02	5.00	622.02	1.18	0.39	1.00
6	3.45	5.00	622.85	5.00	622.85	1.12	0.30	1.00
7	3.37	5.00	623.06	5.00	623.06	1.13	0.35	1.00

## Data Availability

The original contributions presented in this study are included in the article. Further inquiries can be directed to the corresponding author.
